# Choroid and choriocapillaris changes in early-stage Parkinson’s disease: a swept-source optical coherence tomography angiography-based cross-sectional study

**DOI:** 10.1186/s13195-022-01054-z

**Published:** 2022-08-25

**Authors:** Yifan Zhang, Li Yang, Yuzhu Gao, Dan Zhang, Yunhan Tao, Hanyue Xu, Yi Chen, Yanming Xu, Ming Zhang

**Affiliations:** 1 Department of Ophthalmology, Sichuan University West China Hospital, Chengdu, 610041 Sichuan China; 2 Department of Ophthalmology, West China School of Medicine, Sichuan University, 37 Guoxue Lane, Wuhou District, Chengdu, 610041 Sichuan China; 3 Department of Neurology, Sichuan University West China Hospital, 37 Guoxue Lane, Wuhou District, Chengdu, 610041 Sichuan China

**Keywords:** Swept-source optical coherence tomography, Parkinson’s disease, Optical coherence tomography angiography, Choriocapillaris

## Abstract

**Background:**

Parkinson’s disease (PD) is one of the most common neurodegenerative diseases in the aging population. Previous literature has reported thinning of the retinal nerve fiber layer, ganglion cell layer, inner plexiform layer, and photoreceptor layer in PD patients. However, very few studies have used swept-source optical coherence tomography (SS-OCT) to study the choroid and choriocapillaris vascular changes in PD and their correlations with altered contrast sensitivity.

**Methods:**

PD patients and controls were enrolled in the current study. We used a CSV-1000E instrument to assess contrast sensitivity and performed SS-OCT and SS-OCTA to measure outer retinal thickness, choroidal thickness, choriocapillaris flow density, choroidal vascular volume (CVV), and choroidal vascular index (CVI).

**Results:**

One hundred eyes of 52 PD patients and 200 eyes of 100 healthy controls were recruited in the present study. Our study found remarkably impaired contrast sensitivity in PD patients (all *P* < 0.05). Significant thinning of the outer retinal layer and the choroid was appreciated in the PD group compared with the healthy controls (all *P* < 0.05). Choriocapillaris flow density, CVI, and CVV were significantly decreased in PD patients compared with healthy controls (all *P* < 0.05). Contrast sensitivity was weakly associated with outer retina thickness in the 3 mm circular area, with 3 cycles per degree being the most relevant (*r* = 0.535, *P* < 0.001).

**Conclusion:**

Our study indicates that there is a significant decrease in contrast sensitivity, outer retina thickness, choriocapillaris flow density, CVI, and CVV in PD patients. This research has also identified a positive correlation between outer retina thickness and contrast sensitivity.

**Supplementary Information:**

The online version contains supplementary material available at 10.1186/s13195-022-01054-z.

## Introduction

Parkinson’s disease (PD) is one of the most common neurodegenerative diseases in the aging population [1, 2]. Genetic factors, protein misfolding, and oxidative stress have been identified as possible mechanisms of PD [3–5]. However, despite numerous in vitro and in vivo studies, many cases are still considered idiopathic, and their exact pathophysiology remains unknown.

The retina is derived from the optic vesicle of the neuroectoderm [6]. The embryological and anatomical correlation between the eye and the central nervous system makes it a window to the brain [7, 8]. Selective dopaminergic cell loss is the key pathological feature of PD, which is not only observed in the brain but also reported to manifest in the retina [8]. Dopamine receptors are distributed in various layers of the retina, and various studies have reported thinning of the individual retinal layers in PD patients [9–11]. The photoreceptor layer consists of rods and cones that receive dopamine chemical signals during light adaptation and color vision [12, 13]. It is particularly important in PD since impaired contrast sensitivity and color vision are common complaints and the blue cone system is known to be affected in PD [14]. In addition to neurodegeneration, impaired microcirculation regulation and small vessel abnormalities have been proposed as contributing mechanisms of PD [15]. The choroid is a vascular tissue that receives approximately 85% of the retinal blood flow and mainly supplies the outer one third of the layers of the retina [16–18]. Although several studies have reported retinal microvascular impairment observed on optical coherence tomography angiography (OCTA) [19], few have studied the changes in the outer retina layers and choroid circulation.

The past decade has witnessed the rapid development of swept-source optical coherence tomography (SS-OCT) and OCTA technologies. SS-OCT uses a 1050 nm tunable swept laser, which offers a faster scanning speed and deeper penetration under the retinal pigmental epithelium (RPE) [16, 20]. It supplies us with high-resolution images of the choroidal vessels and quantitative data on the microcirculation [21, 22], which allowed us to study the choroid structure and circulation in a more precise fashion. OCTA algorithms were generated based on the analysis of movement signals of erythrocytes on repeated B-scans. It has gained particular attention in clinical practice and scientific research due to its noninvasiveness, fast scanning speed, and quantitative data analysis features.

In the present study, we performed SS-OCT, SS-OCTA, and quantitative analysis in early-stage PD patients. We investigated the structural and functional changes of the outer retina and the choroid in the early stage of PD and discussed the pathophysiological impact of PD on the retina.

## Methods

One hundred eyes of 52 PD patients and 200 eyes of 100 sex-matched, age-matched healthy controls were recruited in the present study. To increase the statistical power of the study, both eyes of subjects were included. The inter-eye correlation was adjusted by generalized estimating equation (GEE). This observational, cross-sectional study was conducted in West China Hospital of Sichuan University, Chengdu, China, from May 2020 to November 2021. The study was approved by the Ethics Committee on Biomedical Research of West China Hospital, No. 2020 (749), and conducted under the Declaration of Helsinki. Written informed consent was obtained from all recruited participants prior to enrollment. This work was supported by the Foundation of Health Commission of Sichuan Province (21PJ023) and China Postdoctoral Science Foundation (2022M710101).

### Neuro-ophthalmic evaluation

The diagnosis and grading of disease severity were conducted using the UK Brain Bank Criteria, Hoehn-Yahr scale (H-Y scale), and Unified Parkinson’s disease rating scale (UPDRS) by a single experienced neurologist (YM.X). Baseline information such as disease duration, medication history, and medication response was recorded. All participants underwent a full neuro-ophthalmic examination, including visual acuity, slit-lamp biomicroscopy, intraocular pressure (IOP), and funduscopic examination. Best-corrected visual acuity (BCVA) values were recorded using a standard Snellen acuity chart and were converted to the logarithm of the minimum angle of resolution (logMAR) scale for statistical analysis. Ophthalmic exclusion criteria were the presence of glaucoma, severe media opacities, uveitis, pathological myopia, macular disease, and optic nerve neuropathy. Neurological exclusion criteria were the presence of other neurological or systemic conditions, including ischemic stroke, Alzheimer’s disease, muscular dystrophy, multiple system atrophy, hypertension, and diabetes mellitus. All controls underwent the same evaluation as PD patients except for the disease diagnosis and grading.

### SS-OCT and SS-OCTA scanning protocol

SS-OCT and SS-OCTA images were obtained with SS-OCT (VG200, SVision Imaging, Ltd., Luoyang, China) with a tunable laser of 1050 nm wavelength and a scanning speed of 200,000 A scan per second [23–26]. The signal strength is measured in real-time during the scanning procedure with the built-in software (Supplement 1). Images with a signal strength greater than 7 were included in the study. The axial optical resolution of SS-OCT is 5 μm, the lateral resolution is 13 μm, and the scanning depth in tissue is 2.7 mm. The axial digital resolution is 2.7 mm/1024 pixels. The scanning mode of SS-OCTA is 6 mm × 6 mm, 512 × 512 pixels, R4. In the selected scanning mode, each B-scan consists of 512 A-scans, and the scanning will be repeated at the same location four times.

### Structural and flow parameters measured by SS-OCTA

**Fig. 1:**
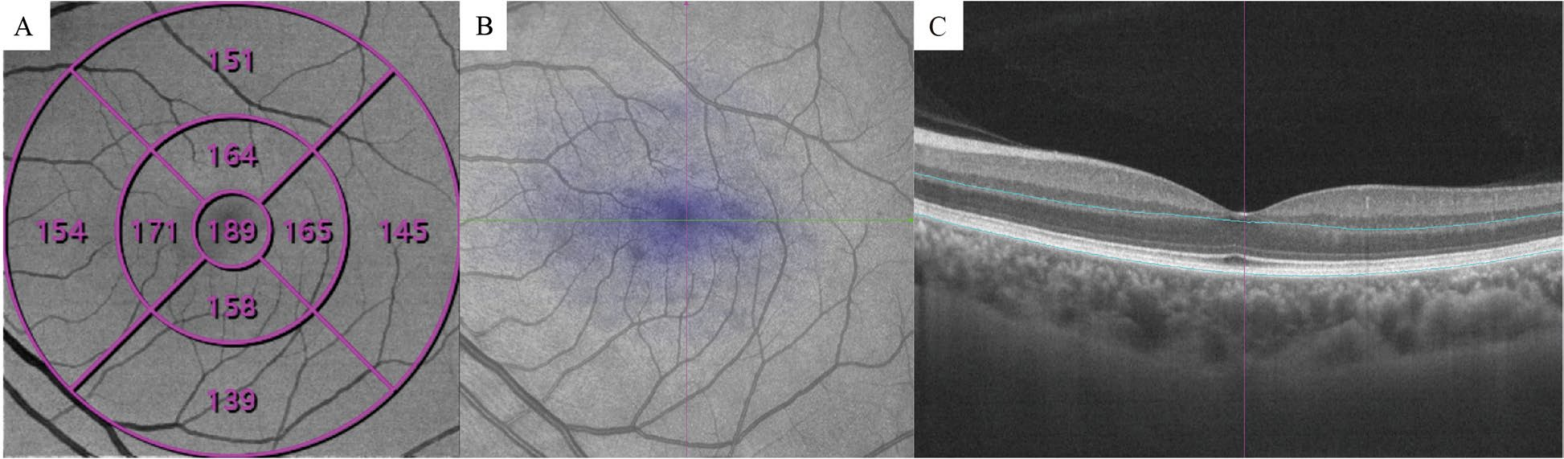
SS-OCTA image of the outer retina. **A** SLO and en face image of the outer retina. Outer retinal thickness was measured with ETDRS, and thickness data of each area was generated by the built-in measurement tool. **B** Thickness map of the outer retina. The blue areas indicate thinning in the layer of interests. **C** The segmentation of the outer retina was marked between the two blue lines

In the present study, outer retinal thickness (ORT) and choroidal thickness (CT) were collected. For data collection, the thickness map was analyzed using the Early Treatment Diabetic Retinopathy Study map (ETDRS) (Fig. 1). ORT refers to the thickness from the lower border of the outer plexiform layer (OPL) to RPE. CT was measured 20 μm blow the Bruch membrane to the choroidoscleral interface. Flow density refers to the area occupied by blood vessels in the layer slice of interests perpendicularly. The presence of a blood vessel is directly indicated by the flow signal of erythrocytes from repeated B-scans [23].

**Fig. 2:**
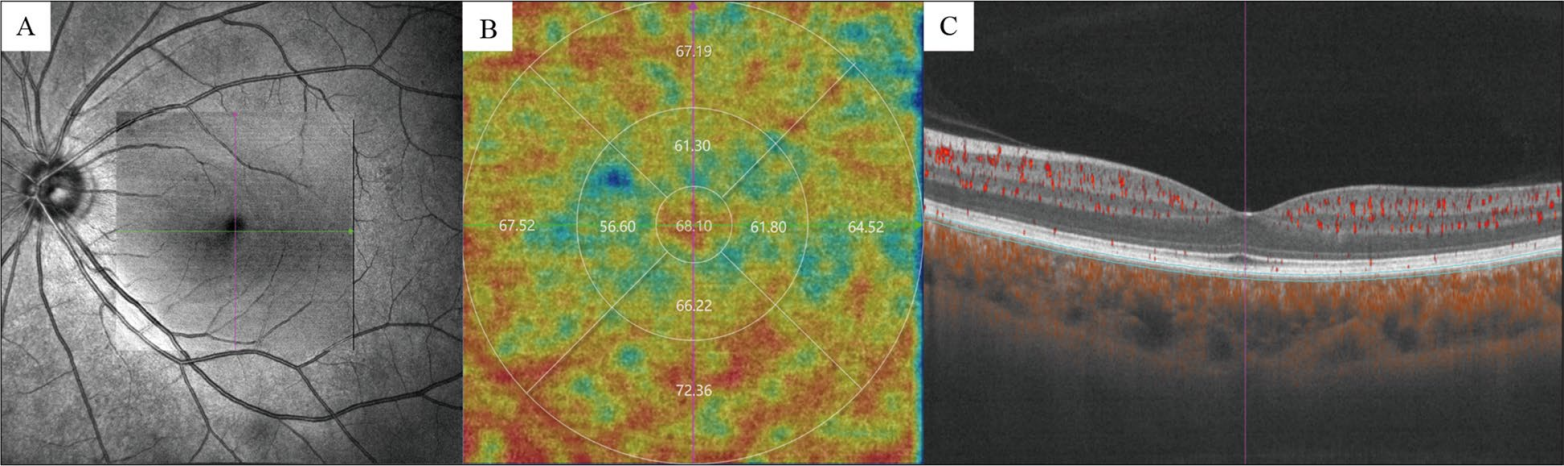
SS-OCTA image of the choriocapillaris. **A** SLO and en face image of the choriocapillaris. **B** Choriocapillaris flow density was measured with ETDRS. **C** Choriocapillaris was defined as 20 μm below Bruch’s membrane, and the automatically segmented choriocapillaris was marked between the blue lines. The retinal flow signal is shown in red, and the choroid flow signal was labeled shown in orange

**Fig. 3:**
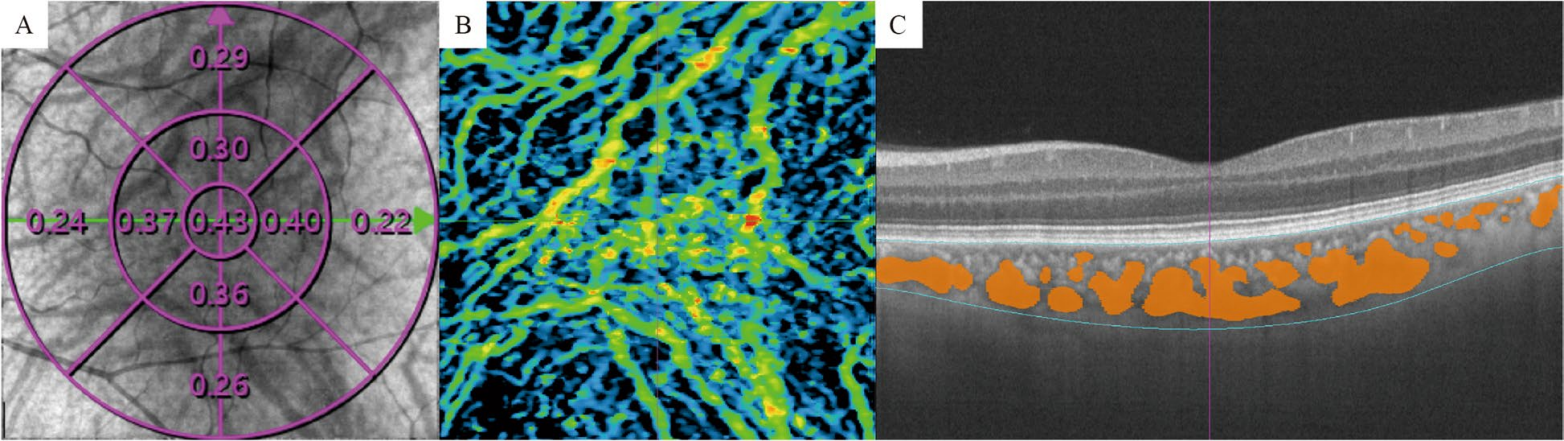
SS-OCTA image of the choroid. **A** SLO and en face image of the choroid. **B** Choroid flow signal demonstrated in the en face image. **C** B- scan of the fovea. Haller’s and Sattler’s layer was marked in orange

The choriocapillaris (CC) is defined and automatically segmented as 20 μm below Bruch’s membrane (Fig. 2). Choroid vessel volume (CVV) is defined as the volume of Haller’s and Sattler’s layers of choroidal vessels. The choroid vessel index (CVI) is defined as the ratio of the volume of Haller’s and Sattler’s layers to the volume of the choroid (Fig. 3). The two parameters were automatically measured by an artificial intelligence-based algorithm that identifies Haller’s and Sattler’s layers on B-scans and reconstructs 3D graphic maps of the medium-diameter and large-diameter choroidal vessel layers. All of the segmentation and measurements were manually checked and corrected by two ophthalmologists (YF.Z and YZ.G). The individual measurements in the areas of interest were documented for statistical analysis.

### Contrast sensitivity

The evaluation of contrast sensitivity was conducted with the CSV-1000E instrument. All subjects were examined with monocular vision at 2.5 m from the chart at four different spatial frequencies (3, 6, 12, and 18 cycles per degree (cpd)). There were 4 rows in the instrument representing each spatial frequency. Each row consists of 17 circular patches with decreasing contrast. All contrast values for each spatial frequency were transformed into a logarithmic scale for statistical analysis.

### Statistical analysis

All thickness and flow measurements are presented as the means ± standard deviations. Student’s *t*-tests were used to compare the ORT, CT, CC flow density, CVI, and CVV of PD patients and healthy controls. Shapiro-Wilk test was performed to test the normality of quantitative data. The baseline difference (age, refractive errors, BCVA, and IOP) between healthy controls and PD patients were tested with Student’s *t*-tests. The differences in binary variables (distribution of gender, smoking status, and alcohol abuse) were tested by a chi-squared test. Our data was adjusted for inter-eye correlation, age, and gender by GEE. The correlations between contrast sensitivity and structural and flow parameters were assessed by Pearson correlation. All statistical analyses were conducted with SPSS software (Windows, Version 26.0, IBM, Armonk, NY, USA). A value of *P* < 0.05 was defined as statistically significant.

## Results

### Baseline characteristics

A total of 100 eyes of 52 PD patients and 200 eyes of 100 healthy controls were enrolled in the present study. Four eyes of PD patients were excluded from the study due to image distortions and insufficient image quality caused by poor eye fixation or media opacity. Baseline demographics of PD patients and healthy controls are indicated in Table 1. There was no significant difference in the age, distribution of sex, BCVA, IOP, spherical refractive errors, alcohol use, or smoking status of the PD group and the control group (all *P* > 0.05). The mean disease duration of our cohort of patients was 2.47 ± 1.51 years, the mean H-Y score was 1.52 ± 0.65, and the mean UPDRS score was 26.32 ± 15.85.

**Table 1:** Baseline demographics and disease characteristics of PD patients and healthy controls

	PD patients (*n* = 52, 100 eyes)	Controls (*n* = 100, 200 eyes)	*P*
Male	26	50	1
Age, years	57.92 ± 8.14	56.63 ± 6.42	0.281
Disease duration, years	2.47 ± 1.51	-	-
SE, D	− 0.28 ± 0.85	− 0.24 ± 0.96	0.88
BCVA, Log MAR	0.93 ± 0.15	0.95 ± 0.17	0.3191
IOP, mm Hg	13.84 ± 3.09	14.26 ± 1.84	0.781
Smoking status	3	7	0.772
Alcohol use	2	3	1
H-Y scale	1.52 ± 0.65	-	-
UPDRS	26.32 ± 15.85	-	-

*SE* spherical equivalent, *BCVA* best-corrected visual acuity, *IOP* intraocular pressure, *H-Y Scale* Hoehn-Yahr scale, *UPDRS* Unified Parkinson’s disease rating scale

### Comparison of structural parameters

A significant decrease in ORT was noted in most of the measured areas (all *P* < 0.05) (Table 2). In quadrant analysis, the outer retina layer revealed remarkable thinning in all four regions of the 6 mm circular region (S, T, I, N, all *P* < 0.05). Significant thinning in the choroid layer of the PD group was also appreciated when compared with healthy controls in most of the measured regions (all *P* < 0.05), except for the superior and nasal quadrants.

**Table 2:** Comparison of outer retinal and choroidal thickness of PD patients and healthy controls (μm)

	Patients	Control	*P*
Outer retinal thickness			
0–1 mm	187.1 ± 14.3	190.9 ± 11.5	0.018
0–3 mm	170.1 ± 12.5	175 ± 8.6	< 0.0001
0–6 mm	151.7 ± 12.8	156.1 ± 7.8	0.002
Superior	152.7 ± 14.7	158.2 ± 8.2	< 0.0001
Temporal	151.9 ± 12.8	156.5 ± 7.8	0.001
Inferior	145.6 ± 12.6	149 ± 8	0.012
Nasal	152.8 ± 13.1	156.5 ± 8.9	0.009
Choroidal thickness			
0–1 mm	313.3 ± 84.3	333.5 ± 76.3	0.049
0–3 mm	306.4 ± 81.6	326.2 ± 71.9	0.043
0–6 mm	289.9 ± 76.9	307.8 ± 63.9	0.044
Superior	314.3 ± 83	328.9 ± 64.9	0.15
Temporal	291.2 ± 79.6	313.7 ± 70.7	0.019
Inferior	290.8 ± 79.5	313.1 ± 73.2	0.023
Nasal	260.6 ± 86.4	272.6 ± 74.4	0.238

### Comparison of flow parameters

A significant decrease in CC flow density was observed in the 1 mm, 3 mm, and 6 mm circular regions (all *P* < 0.001), Table 3. However, in quadrant analysis, significant differences were only appreciated in the superior and inferior sectors (*P* = 0.001, < 0.0001). However, the differences between the temporal and nasal sectors were not statistically significant (*P* = 0.062, 0.082). In the choroid layer, there was a significant reduction in the choroidal flow of Haller’s and Sattler’s layers in PD patients when analyzed based on CVI and CVV in most of the measured regions, except for the 1 mm circular area and the nasal sector (*P*>0.05).

**Table 3:** Comparison of choriocapillaris flow density, CVI, and CVV of PD patients and healthy controls

	Patients	Control	*P*
CC flow density (%)			
0–1 mm	70.48 ± 10.11	76.29 ± 10.98	< 0.0001
0–3 mm	73.64 ± 5.89	76.95 ± 6.59	< 0.0001
0–6 mm	76.17 ± 3.68	78.26 ± 5.04	< 0.0001
Superior	77.45 ± 4.15	79.45 ± 5.28	0.001
Temporal	74.51 ± 3.14	75.77 ± 2.81	0.062
Inferior	77.18 ± 3.77	79.49 ± 5.41	< 0.0001
Nasal	76.32 ± 4.29	77.35 ± 5.45	0.082
CVI		0 ± 0	
0–1 mm	0.38 ± 0.13	0.37 ± 0.07	0.871
0–3 mm	0.34 ± 0.12	0.37 ± 0.07	0.03
0–6 mm	0.31 ± 0.11	0.35 ± 0.07	0.003
Superior	0.31 ± 0.12	0.36 ± 0.08	0.01
Temporal	0.3 ± 0.11	0.34 ± 0.08	0.006
Inferior	0.31 ± 0.12	0.36 ± 0.08	0.001
Nasal	0.31 ± 0.12	0.34 ± 0.09	0.127
CVV (mm^3^)		0 ± 0	
0–1 mm	0.11 ± 0.05	0.11 ± 0.03	0.94
0–3 mm	0.85 ± 0.4	0.91 ± 0.37	0.151
0–6 mm	2.89 ± 1.43	3.27 ± 1.12	0.023
Superior	0.74 ± 0.37	0.87 ± 0.31	0.002
Temporal	0.68 ± 0.34	0.78 ± 0.29	0.008
Inferior	0.71 ± 0.38	0.82 ± 0.33	0.017
Nasal	0.67 ± 0.39	0.69 ± 0.33	0.485

*CC* choriocapillaris, *CVI* choroidal vascular index, *CVV* choroidal vascular volume

### Comparison of contrast sensitivity and its correlation with SS-OCT variables

**Fig. 4:**
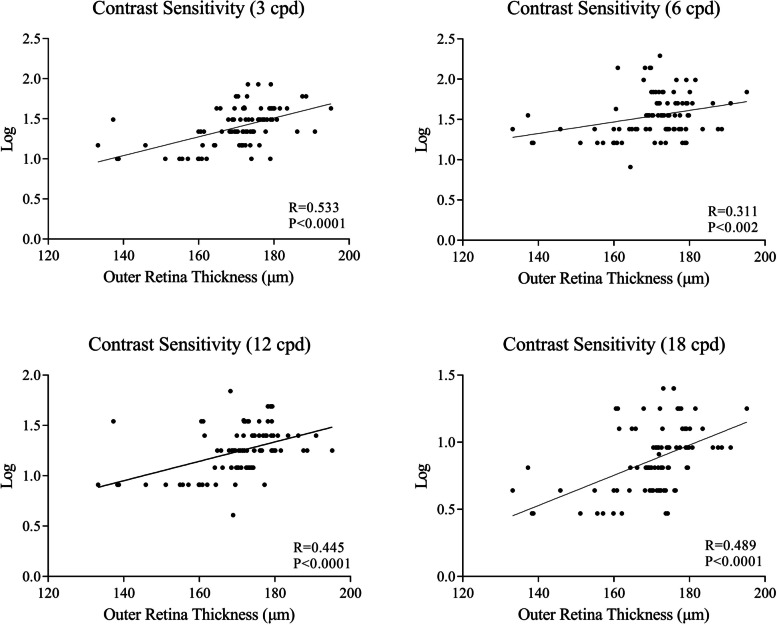
Correlations of contrast sensitivity and outer retinal thickness at 3, 6, 12, and 18 cycles per degree

Contrast sensitivity was significantly decreased at 3, 6, 12, and 18 cpd compared with healthy controls (all *P* < 0.05, Table 4). Contrast sensitivity was weakly associated with ORT in the 3 mm circular area, with 3 cycles per degree being the most relevant (*r* = 0.535, *P* < 0.001, *r* = 0.458, *P* = 0.003, *r* = 0.481, *P* = 0.002 in the 1 mm, 3 mm, and 6 mm circular areas, respectively) (Fig. 4). We did not appreciate any associations between CC flow density, CVI, CVV, and contrast sensitivity.

**Table 4:** Contrast sensitivity test in PD patients and healthy controls

	PD patients	Controls	*P*
cpd 3	1.39 ± 0.23	1.56 ± 0.22	< 0.001
cpd 6	1.54 ± 0.26	1.71 ± 0.21	< 0.001
cpd 12	1.00 ± 0.2	1.25 ± 0.32	< 0.001
cpd 18	0.87 ± 0.24	1.02 ± 0.18	< 0.001

*cpd* cycles per degree

## Discussion

In this cross-sectional study, we investigated the structural and functional changes of the outer retina and the choroid in the early stage of PD using SS-OCT and SS-OCTA. Compared to our previously published article [23], we focused on the outer retina and the choroid circulation change in PD patients. By expanding the sample size, we found decreased contrast sensitivity, thinning of the outer retina layer, and decreased choriocapillaris flow density, CVV, and CVI in early-stage Parkinson’s disease.

It is a widely held view that contrast sensitivity and color vision is impaired in PD [27, 28]. Jackson et al. reported impaired contrast sensitivity, reduced rhythmic light-adapted ERG, and visual acuity in D4 receptor knockout mouse models. In particular, D4 receptors are preferentially expressed in cone photoreceptors and participate in rod-cone coupling, the transmission of signals, and gene expression [29]. In line with previous studies, a prominent decline in contrast sensitivity was also appreciated in our cohort of patients.

### The correlation between functional and structural changes of the retina in PD

Multiple pathways are involved in the processing of visual perception signals, which may offer some explanations for the ophthalmic symptoms observed in PD [30, 31]. The midget-parvocellular (P) and parasol-magnocellular (M) visual pathways are the two main pathways that serve conscious visual sensory, especially red-green color discrimination and spatial contrast sensitivity [32–34]. The midget retinal ganglion cells and cone photoreceptors of P pathway, which is responsible for high spatial signal delivery and red-green color vision, are primarily located in the macular region [35]. Visual information generated by cone photoreceptors after light stimulation is transmitted via bipolar cells to midget cells, which then project to the parvocellular layer of the lateral geniculate nucleus (LGN) [17, 36]. Parasol cells, on the other hand, are mostly found in the periphery and are responsible for motion detection and low spatial frequency contrast sensitivity [33, 35, 37]. Axons from parasol cells merge into the optic nerve via the superior, nasal, and inferior sectors, which ultimately project to the magnocellular layers of the LGN and the visual cortex [32, 38]. However, the involved pathway in Parkinson’s disease requires further investigation. According to some studies, the P-pathway appears to be preferentially involved in PD, whereas others claim that neurodegenerative diseases generally affect low-frequency spatial contrast sensitivity [30, 31, 39]. Our study found a decrease in contrast sensitivity across all the spatial frequencies, which is related to the ORT. It might be difficult to explain this finding based on OCT studies since multiple pathways might be compromised in PD, which eventually leads to the observed clinical symptoms. In addition, the involvement of the CNS, such as the LGN and visual cortex, cannot be neglected [30, 40]. Cellular and disease model research remains to be finished to offer some explanations for the functional and morphological changes of the retina in PD.

### The application of recent development in SS-OCT in neurodegenerative disease

In recent decades, OCT- and OCTA-based neurodegenerative disease studies have generated hundreds of publications, even though the results are sometimes contradictory. Roth et al. reported that thinning of the photoreceptor layer and outer nuclear layer is associated with impaired color vision and disease severity [13]. However, they did not appreciate any change in the thickness of the RNFL or GCL. Another study carried out by Polo et al. demonstrated a correlation between contrast sensitivity and GCL thickness assessed by SD-OCT [41]. However, they did not disclose any of the findings of the outer retina in their study. Our study found a significant decrease in the outer retina; however, changes in the RNFL and GCL+IPL layers were not evident in our cohort of patients. This discrepancy could be attributed to several reasons: (1) previous studies were conducted with time-domain OCT or SD-OCT, and in some studies, manual segmentation was performed. In contrast, the present study was conducted with a high-resolution SS-OCT device with a scanning speed of 200,000 A-scans per second, which is greater than other commercially used SS-OCT devices. As far as OCTA is concerned, the higher speed of SS-OCT brings some unique advantages and more flexibility for non-invasive retina imaging. Since a higher speed allows acquiring more A-scans within the same amount of time and therefore allows a denser sampling on the retina, which is necessary to capture the detail of capillaries and blood flow. A typical OCTA scan protocol on an SD-OCT generates 256 × 256 pixels or 384 × 384 pixels OCTA images, whereas the 200 kHz SS-OCT employed in this study has standard protocols of 512 × 512 pixels acquired within a similar time. SS-OCT has two additional advantages over SD-OCT when imaging choroidal layers. First, SS-OCT has significantly reduced fringes wash-out effect and sensitivity roll-off compared to SD-OCT and thus the sensitivity remains flat in a relatively long-range whereas SD-OCT drops quickly over depth [42, 43]. Second, SS-OCTs typically operate at a longer wavelength than commercially available SD-OCTs [44, 45]. The long-wavelength of 1050 nm enables deeper in-tissue penetration and less signal decay under the RPE layer, which is particularly favored in studying retinal and choroid circulation [21, 22]. (2) Although previous studies have reported their patients as having early-stage PD, we recognize that there is a difference in patient selection. The mean age, disease duration, and disease severity in our cohort of patients might be younger and lower. (3) Dopaminergic cells only contribute to a small percentage of the GCL+IPL layer cell bodies [9, 46]. The mild degeneration of the GCLs and a slight decrease in thickness might be insignificant to detect by SS-OCT at this stage of the disease.

### Vascular factors in the pathogenesis of PD

The contribution of vascular factors to retinal degeneration in PD has not reached a consensus. Postmortem studies conducted by Schwartz et al. and Mastaglia et al. did not favor a vascular cause in the development of PD [15, 47]. However, contrary to those studies, Holst et al. claimed that the 5-year risk of developing PD is associated with high white matter abnormalities observed on MRI, which indicates that vascular factors might be attributed to the etiology of PD [48]. Moreover, the involvement of arterial vessels has been reported based on immunocytochemistry and living retinal imaging studies. Price at el. demonstrated α-syn-GFP deposition around retinal arterial vessels and in the interstitium of mouse models [49]. This also agrees with the study conducted by Kwapong et al., which indicated that decreased superficial retinal capillary plexus is associated with thinning of the GCL+IPL layer [19]. In light of the similarity and embryological origin of the retina and the central nervous system, it is reasonable to postulate that the retina shares similar pathology.

However, little is known about the involvement of the choroid and choriocapillaris changes in PD patients. We found only three studies reporting choroid thickness changes in PD, and none of them used SS-OCTA to assess vascular parameters. Garcia-Martin et al. reported thickening of the peripapillary choroid in all sectors in PD patients compared with controls [50]. Their conclusion was further supported by Satue et al., who reported thickening of the choroidal layer both in the macular and peripapillary areas observed by SS-OCT [51]. However, in contrast to those findings, Eraslan et al. performed enhanced depth imaging optical coherence tomography (EDI-OCT) on 44 eyes of PD patients, and their research favors volume loss of the choroid layer [52].

To the best of our knowledge, we are the first study using SS-OCTA to study choroid circulation in PD patients. Our study indicates that there is a significant decrease in the flow density of the choriocapillaris, CVI, and CVV in early-stage PD patients. We postulate that choroid hypoperfusion might be the explanation for choroid volume loss, the reasons are as follows: (1) the choroid receives approximately 85% of the retinal blood flow, which is subject to ischemic events and oxidative stress. In reviewing the literature on neurodegenerative diseases, amyloid deposits were identified in the choriocapillaris, retinal, and choroidal vasculature in an Alzheimer’s disease mouse model [53]. In accordance with this evidence, an OCTA-based study indicated that the choroidal flow rate is significantly slower in Alzheimer’s disease patients, which might offer an explanation for the failure of amyloid deposit degradation in a mouse model [54]. Promising evidence has also been reported from living retina imaging of a PD mouse model, which reveals remarkable retinal α-syn depositions around retinal arteries [55]. Whether a similar distribution pattern of α-syn depositions in the choroid circulation could be observed in PD relies on further histopathological evidence. (2) The autonomous nervous system is known to be affected in PD [56]. However, choroid circulation is regulated by sympathetic and parasympathetic innervation. The Braak hypothesis indicates that PD patients exhibit Lewy body pathology in the dorsal motor nucleus of the glossopharyngeal and peripheral nerves [57]. Whether the same pathology exists in the regulation of choroid and retinal perfusion remains an open discussion. (3) The choroid supports and nourishes the outer layers of the retina. Photoreceptors are sensitive to light injury and oxygen-induced stress due to their extensive metabolism and rapid turnover rate [17, 18]. The diminished choroid perfusion might potentially affect its ability to clear free radicals and metabolic toxins [57], which might contribute, to some degree, to the impaired sensitivity in PD patients. It is somewhat surprising that the relationship between ORT, CC flow density, CVI, and CVV in this study was not significant. A possible explanation for these results may be the lack of adequate samples, and the relationship might be revealed in larger-scale studies. Another probable explanation for this is that the choroid is a highly variable structure, and the association between contrast sensitivity and the outer retina might not be a simple linear relationship but rather much more complicated.

### Challenges in performing ophthalmic imaging research in PD

Unique disease features of PD have made it challenging sometimes to obtain high-quality images. First, pupillary autonomic dysfunction has been reported to be one of the symptoms of PD [58], which is consistent with what we observed from the pupil camera (Supplement 2). In some patients, the relative restricted pupil size and spontaneous changes in pupil diameter might not affect the image quality of the macular area; however, the brightness of the peripheral image might be compromised sometimes (Supplement 3). Since the pupil controls the overall light volume shed into the retina, dilating eye drops could be considered before obtaining OCTA images after careful ophthalmic evaluation and exclusion of contraindication to achieve optimal peripheral imaging quality. Second, the excluded images with poor image quality and image acquisition failure warrant discussions for the OCT manufacturers and OCTA analyzing algorithm development. The landmark of PD is its motor symptoms, resting tremors of 4 Hz–6 Hz, which might vary after medical intervention [59]. Even though the current device is equipped with an eye-tracking software to minimize artifacts and distortions, however, the software is not perfect in every disease setting. The most frequent reason for exclusion is image distortion in patients with significant motor symptoms, which is usually more prominent in a smaller scanning view (Supplement 4). Since the eye-tracking algorithm was built mostly based on movement patterns of healthy controls and general ophthalmic patients, the eye movement caused by uncontrolled resting tremors usually exhibits a higher frequency compared to voluntary eye movements, which might be the primary reason for failing to track and restore OCTA images. The exclusion images and acquisition failure in PD needs to be studied case by case for eye-tracking program optimization, which could be tailored to neuro-ophthalmology patient population.

### Limitations

There are several limitations of our study that should be taken into consideration when interpreting the results. First, our study was conducted with small sample size, and the results need to be verified on larger scale. Another limitation of our study is the relatively small area of interest focusing on the posterior pole. Even though the largest scanning area of the device we are using is 12 × 12 mm^2^, the image quality in the peripheral area was not sufficient for data analysis due to the small pupillary diameter (Supplement 3). Ideally, dilating eye drops could be administered before image acquisition.

### Future directions

The use of SS-OCT and SS-OCTA to study PD is still in its nascent stages. Some innovative technologies could potentially provide us with future directions and promising prospects. For example, adaptive optics (AO) technology and living retinal microscopy have been used to study diabetic retinopathy and other diseases in ophthalmology [60]. Direct visualization of the retina and retinal vasculature in vivo could allow us to study retinal microcirculation and the cellular structures of the retina in layers, which could detect pathological changes at the cellular level. The application of AO is of great importance in PD since the dysfunction of cone and rod photoreceptors has been reported in PD disease models [61]. Another promising research field is the use of mathematical modeling and combined artificial intelligence to reconstruct the ocular blood flow, hemodynamics, and oxygen transport system in the eye [55]. The collaboration of physics and mathematical models might potentially provide a solution to the complexity and difficulty of studying human ocular blood flow.

## Conclusion

To the best of our knowledge, we are the first study using SS-OCT and SS-OCTA to study choroid circulation in PD patients. This thesis has provided deeper insight into the contribution of vascular factors in PD. Our study indicates that there is a significant decrease in contrast sensitivity, ORT, flow density of choriocapillaris, CVI, and CVV in PD patients. This research has also identified a positive correlation between ORT and contrast sensitivity.

## Supplementary Information

**Additional file 1.** The formula of signal strength measurement and the algorithm used in segmentation of the choroidal vessels in Haller's and Sattler's layers .

**Additional file 2.** Pupil size changes observed on the pupil camera.

**Additional file 3.** The effect of pupil size on image quality. 5A. SLO image of an included subject with normal pupil size. The image exhibits sufficient brightness in the peripheral areas. Figure 5B. SLO image of an excluded subject. Although the imaging of the macula area is adequate, the peripheral areas showed dark regions (blue triangles). Figure 5C. Another excluded image with a severely constricted pupil and cataract, the image shows insufficient brightness and poor visualization of the periphery.

**Additional file 4.** Demonstration of the excluded images with distortions. 6A. 3×3mm SS-OCTA image of PD patients. The OCTA image showed distortion when failing to align the flow signal in the same plane (blue star) and artifacts were created during severe resting tremors (orange triangle). 6B. 6×6 mm SS-OCTA image of PD patients. The distortion was smaller compared to 3×3mm (blue triangle), and lines of artifacts were still visible (orange triangle). 6C. 12×12mm SS-OCTA image of a PD patient, the distortion was almost unnoticeable at the image size, artifacts were labeled with an orange triangle.

## Data Availability

The data used to support the findings of this study are available from the corresponding author upon request.

## Abbreviations

PD: Parkinson disease
SS-OCT: Swept-source optical tomography
SS-OCTA: Swept-source optical tomography angiography
CVI: Choroidal vascular index
CVV: Choroidal vascular volume
RNFL: Retinal nerve fiber layer
GCL: Ganglion cell layer
IPL: Inner plexiform layer
ORT: Outer retinal thickness
RPE: Retinal pigmental epithelium
