# Supplementary material for: Choroid and choriocapillaris changes in early-stage Parkinson’s disease: a swept-source optical coherence tomography angiography-based cross-sectional study
Source: Alzheimers Res Ther. 2022 Aug 25;14:116. doi: 10.1186/s13195-022-01054-z (PMC9404633; doi:10.1186/s13195-022-01054-z)
Supplement: Supplementary file 1 — Additional file 1. The formula of signal strength measurement and the algorithm used in segmentation of the choroidal vessels in Haller's and Sattler's layers . [file 13195_2022_1054_MOESM1_ESM.docx]

1. SVision VG200 SS-OCT image signal strength measurement

The signal strength represents the overall intensity of the imaged feature.

The formula provided by the manufacturer for calculating the signal strength are as follows:

OCT absolute_signal_strength is signal intensity in linear = (OCT signal in linear - background)^2^.

Displayed in UI is relative_signal_strength = 5×log10(absolute_signal_strength×depth_mm / 5.4).

1. The algorithm that was used in segmentation of the choroidal vessels in Haller's and Sattler's layers.

Choroidal vessel detection algorithm is a convolutional neural network based algorithm derived from a popular architecture named UNet, with an encoder path that looks at features of increasing lower resolutions, and a decoder path that does the opposite. A bypass link connects the two at each resolution, preserve feature's spatial information that would have been compromised during repeated pooling, enabling it to perform accurate semantic segmentation down to pixel level. Preprocessing steps are added before feeding the oct images to the network to faciliate easier convergence, and parameters and configuratoins of the network are customized to best serve the purpose of choroidal vessel detection.
