# Supplementary figures and images for: Choroid and choriocapillaris changes in early-stage Parkinson’s disease: a swept-source optical coherence tomography angiography-based cross-sectional study
Source: Alzheimers Res Ther. 2022 Aug 25;14:116. doi: 10.1186/s13195-022-01054-z (PMC9404633; doi:10.1186/s13195-022-01054-z)

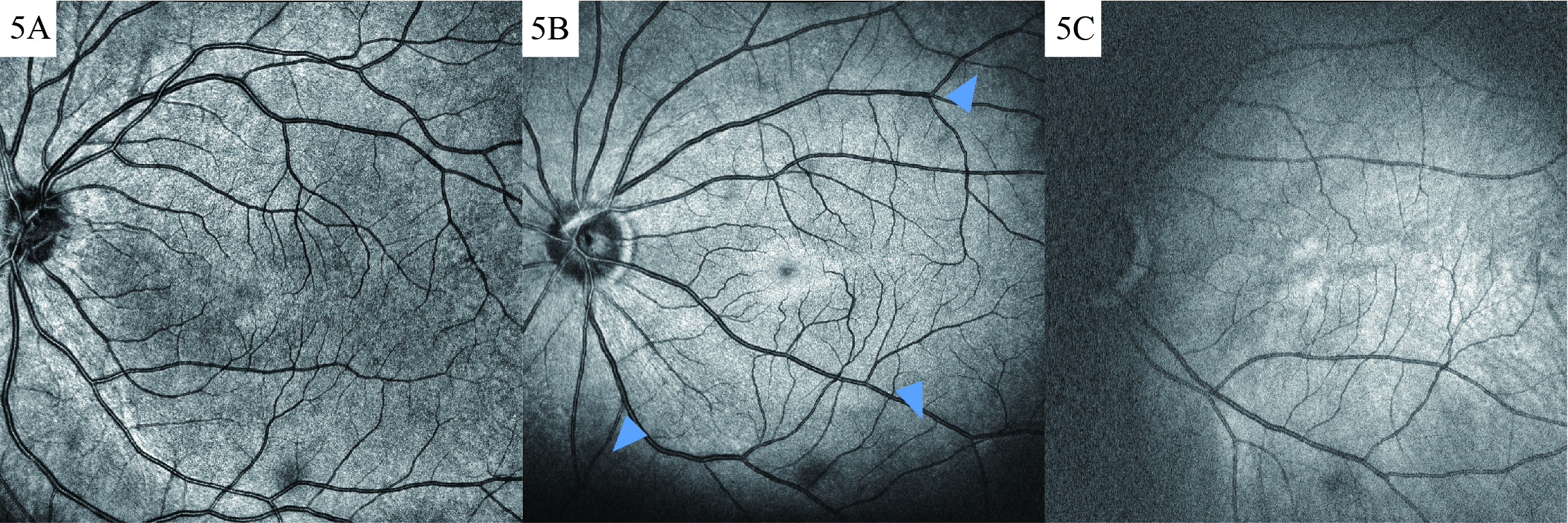

Supplement: Supplementary file 3 — Additional file 3. The effect of pupil size on image quality. 5A. SLO image of an included subject with normal pupil size. The image exhibits sufficient brightness in the peripheral areas. Figure 5B. SLO image of an excluded subject. Although the imaging of the macula area is adequate, the peripheral areas showed dark regions (blue triangles). Figure 5C. Another excluded image with a severely constricted pupil and cataract, the image shows insufficient brightness and poor visualization of the periphery. [file 13195_2022_1054_MOESM3_ESM.jpg]

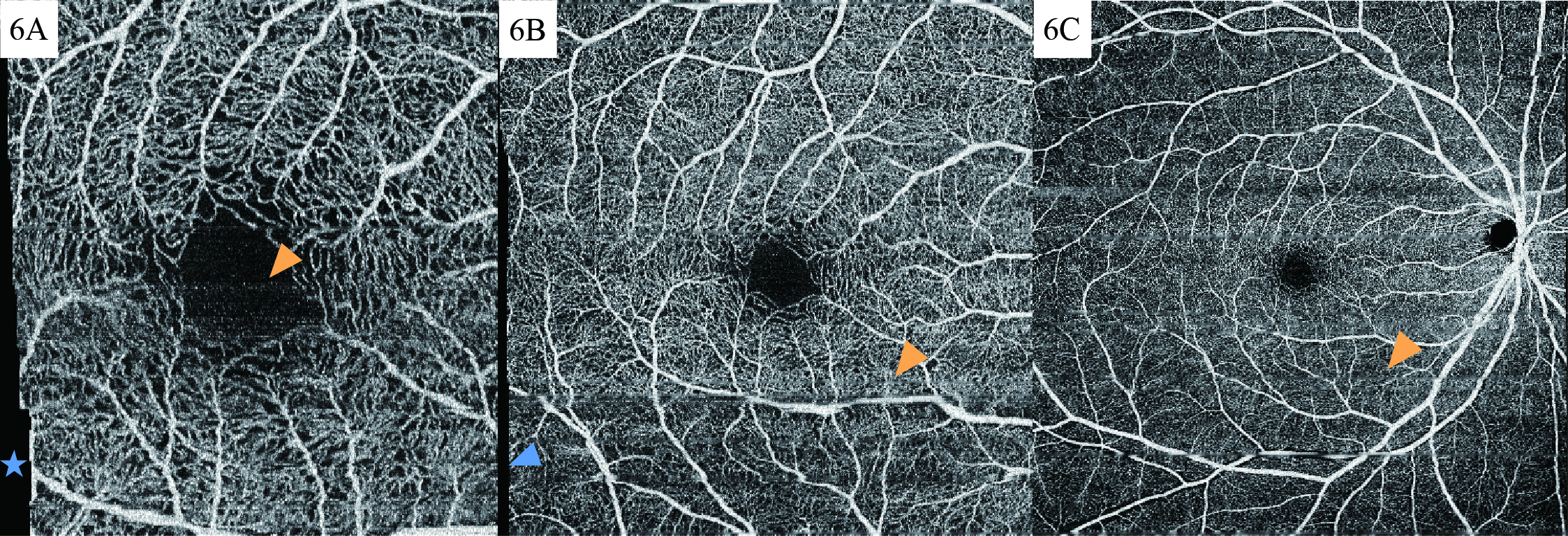

Supplement: Supplementary file 4 — Additional file 4. Demonstration of the excluded images with distortions. 6A. 3×3mm SS-OCTA image of PD patients. The OCTA image showed distortion when failing to align the flow signal in the same plane (blue star) and artifacts were created during severe resting tremors (orange triangle). 6B. 6×6 mm SS-OCTA image of PD patients. The distortion was smaller compared to 3×3mm (blue triangle), and lines of artifacts were still visible (orange triangle). 6C. 12×12mm SS-OCTA image of a PD patient, the distortion was almost unnoticeable at the image size, artifacts were labeled with an orange triangle. [file 13195_2022_1054_MOESM4_ESM.jpg]
